# Personal experience, posttraumatic symptomatology, and meaning in life during the first months of the COVID-19 pandemic

**DOI:** 10.1007/s12144-021-02487-9

**Published:** 2021-12-03

**Authors:** A. Y. Arredondo, B. Caparrós

**Affiliations:** Psychology Department, University of Girona, Plaça Sant Domènec, 9. Campus Barri Vell, 17004 Girona, Spain

**Keywords:** COVID-19, Pandemic, PTSD, Meaning in life

## Abstract

The traumatic subjective distress and personal meaning in life were examined in the context of the first months of the COVID-19 pandemic sanitary crisis and home lockdown. Method: A total of 543 participants answered an online survey that included questions about the individual characteristics of the pandemic experience, the Impact of Event Scale-Revised, and the Personal Meaning Profile-Brief. Results: Nearly all of life impaired areas, having the suspicion of being ill with COVID-19, having lost a close person to this virus, and having been accompanied during the lockdown were experiences associated with higher PTSD symptoms. Posttraumatic symptomatology was inversely correlated with areas of meaning in life. Lastly, a higher number of affected areas and a negative subjective lockdown circumstance explained greater total PTSD symptoms. Conclusion: Specific pandemic experiences and lockdown circumstances affected the presence of posttraumatic symptoms. The personal meaning of life seems to be involved in the process of less adverse traumatic consequences.

Since the COVID-19 pandemic began spreading worldwide, individuals have been affected not only by the disease (and its direct implications, such as the physical sequelae; Fiani et al., 2020; Hays, 2020), but by a disturbing experience that has impacted them psychologically (Balluerka et al., 2020; Burhamah et al., 2020; Sher, 2020; Xiong et al., 2020; Varshney et al., 2020). While the pandemic has changed everyone's life, the impact on individuals may depend on their particular experiences, i.e., sudden life changes or their proximity to the virus. Additionally, the impositions of public health measures that infringe on personal freedoms, such as home lockdown and its specific conditions, gave rise to adverse mental effects (Ammar et al., 2020; Gualano et al., 2020; Pfefferbaum & North, 2020; Rehman et al., 2020; Vázquez et al., 2021). The massive-scale social isolation and its derived sense of loneliness and disconnection have substantially and negatively impacted life satisfaction and well-being (Bzdok & Dunbar, 2020) and increased the risk of mental decline (Killgore et al., 2020). These outcomes are alarming due to the links of isolation to other adverse clinical outcomes, such as suicidal behavior (De Berardis et al., 2018). Also, the involvement of sensory perception in emotional processes has been shown to be a crucial factor to consider in traumatic situations (Serafini et al., 2016). At large, this pandemic has been an unexpected and unprecedented mental health hazard in which fear, helplessness, and uncertainty stand out. In addition, both new findings on SARS-CoV-2 and knowledge about previous infectious disease epidemics point to mental health risk factors, and posttraumatic stress disorder (PTSD) correlates (Boyraz & Legros, 2020; Karatzias et al., 2020).

While the negative impact of the pandemic is unquestionable, the possibility of positive experiences after distressing situations exists. Individuals can habituate, learn, cope, deal with difficulties, and even display beneficial results like appreciating life itself (Janoff-Bulman & Berger, 2000). Personal values, ideals, and perspectives make individuals’ life meaningful (Wong, 2012). The experience of adversity has been linked with the personal meaning in life. A deep wound caused by trauma may comprise the meaningfulness towards goals, profession, connections, and individual evolvement (Nader, 2006), and trauma-related distress may stimulate questioning and meaning-making (De Castella & Simmonds, 2013). Thus, this wound’s existential considerations are not necessarily negative and might lead to reflection, introspection, and life revaluation. Moreover, previous findings suggest that individuals with higher perceived meaning have fewer PTSD symptoms (Aiena et al., 2016).

Studies on the COVID-19 pandemic show, on the one hand, that meaning in life buffered anxiety and nonproductive thinking despite unpredictable threats (Trzebiński et al., 2020). On the other hand, meaning in life has acted as a risk factor for adverse psychological outcomes in people’s experience of boredom (Chao et al., 2020). Despite the obvious psychological impact of the pandemic, we do not know much about the particularities of such an experience, nor about its possible association with post-traumatic symptoms and meaning in life. By exploring these relationships, we intended to achieve a greater understanding of the pandemic-related distress and its connection with the personal sense of purpose.

## Objectives

This study aims to measure the relationships among the pandemic’s personal experience (i.e., lockdown characteristics, possible proximity to the virus, and life affectations), the presence of trauma-related symptoms, and the personal meaning in life in persons affected by the COVID-19 pandemic. The specific objectives are to analyze the possible associations between 1) features of the experience of the pandemic and PTSD symptoms, 2) features of the personal experience of the pandemic and personal meaning in life, and 3) PTSD symptoms and meaning in life. Lastly, 4) to explore which of the studied variables explain higher PTSD symptoms.

We expected that a greater proximity to the virus and more severe home confinement circumstances would be associated with higher PTSD symptoms. Based on previous findings, we hypothesized that the personal meaning in life would be inversely correlated with PTSD symptomatology. Finally, we expected more severe pandemic experiences and lower meaning in life would explain a higher total PTSD symptomatology.

## Methodology

### Participants

A total of 543 participants were included in this study. The majority were female (*n* = 478, 78.8%), and 21.2% were male. The average age was 36.88 years (*SD* = 12.59), and ages ranged from 18 to 73. When dividing into age groups, 68% corresponded to early adulthood (18 to 39 years old), 30.6% to mid-adulthood (40 to 64), and 1.5% to late adulthood (from 65 onwards). Most participants lived in the American continent (*n* = 458, 84.3%), 15.3% lived in Europe, and 0.4% in Oceania. The survey was answered by people from 28 different countries (including Spain, Argentina, United States, Colombia, and Guatemala); the majority of them were Mexican residents (*n* = 369, 68%).

### Measures

#### Basic and Pandemic-Related Information

We created an ad-hoc questionnaire for collecting basic information (age, sex, and country of residence). In addition, it examined details about the individual experience of the pandemic. It included a list of possible life-affected areas (work, academic, economic, social relationships, couple relationships, mental health, and physical health). Moreover, respondents indicated their proximity to the virus: If they knew one or more close persons who have died as a consequence of the coronavirus, if they suspected being ill with COVID-19 but were not diagnosed, if they were positively ill due to the virus (mildly, moderately or seriously), and if they had associated sequelae. Lastly, participants reported the number of days they were in confinement, a general assessment of the confinement experience, from 1 (*very negative*) to 5 (*very positive*), if they were confined alone or accompanied, and a corresponding assessment rating from 1 (*very negative*) to 5 (*very positive*).

#### Traumatic Symptoms

For assessing the subjective distress caused by the traumatic event, we used the Impact of Event Scale-Revised (IES-R; Weiss & Marmar, 1997) spanish version (Baguena et al., 2001). It includes 22 items rated from 0 (*not at all*) to 4 (*extremely*) on the degree of distress experienced during the last week. The IES-R yields a total score (from 0 to 88), which is divided into normal (0–23), mild (24–32), moderate (33–36), and severe psychological impact (> 37). Subscale scores can be calculated for Intrusion (8 items), Avoidance (8 items), and Hyperarousal (6 items). Internal consistency values ranged 0.87- 0.94 for Intrusion, 0.84- 0.87 for Avoidance, and 0.79-0.91 for Hyperarousal (Creamer et al., 2003; Weiss & Marmar, 1997). For this study, the total scale’s consistency value was 0.90.

#### Meaning in Life

The Personal Meaning Profile-Brief (PMP-B; McDonald et al., 2012) Spanish version (Carreño et al., 2020). This 21-item scale assesses meaning in life through seven sources: Relationship (having friends, being liked and trusted by others), intimacy (mutually satisfying relationships), Achievement (striving for and attaining life goals), Self-acceptance (accepting personal limitations and suffering), Self-transcendence (contributing to society), Fair treatment (perceiving fairness from society and life), and Religion (seeking to please God). Items are rated on a Likert scale from 1 (*not at all*) to 7 (*a great deal*). Total scores range from 21 to 147, and higher scores indicate more success in approximating an ideally meaningful life. Alphas for the Spanish version ranged between 0.64 and 0.91 (Carreño et al., 2020). For this study, the total scale consistency value was 0.88.

### Procedure

The invitation to participate in this study was spread by social networks (WhatsApp, Facebook, and Instagram) using a snowball sampling method during the pandemic’s initial stage (June to the first week of August 2020). The inclusion criteria comprised persons that had experienced the COVID-19 pandemic and related confinement measures. When first entering the survey page, the study objectives and the questionnaire’s estimated duration were communicated. If individuals consented and confirmed being over 18 years old, they were directed to the survey. Respondents answered an online and anonymous self-administered questionnaire in Spanish language and, upon completion, were asked to invite their acquaintances to participate. Lastly, statistical analyses were developed with SPSS v26.

## Results

### Descriptive Results

The areas of life most frequently affected were social and work (Table 1). The most common COVID-19 related experiences were knowing someone who had the virus, knowing someone who died due to the virus, and suspecting they had coronavirus. The average days of confinement were 88.8 days (*SD* = 34.38). Most participants were locked down with another person/s (*n* = 500, 92.1%), and only 7.9% by themselves. Participants rated on average 3.25 (*SD* = 1.00) their confinement experience and 4.06 (*SD* = 0.91) their experience of being alone or with someone else. Additionally, individuals who were alone (*M* = 3.70, *SD* = 1.08), comparing to those who were in the company of others (*M* = 3.33, *SD* = 0.99), reported a significantly better experience during home lockdown (*t* = 2.308, *p* = 0.021).

**Table 1:** Frequency and percentages of affected life areas and personal proximity to COVID-19

Areas of life affected by COVID-19 crisis	***N***	***%***
Work	304	56.0
Academic	133	24.5
Economic	274	50.5
Social relationships	353	65.0
Couple relationship	149	27.4
Mental health	260	47.9
Physical health	196	36.1
COVID-19-related experiences	***N***	***%***
I know someone who has the virus	367	67.7
One or more close persons have died as a result of the virus	97	17.9
I suspect I have the virus, but I have not been diagnosed	91	16.8
I was/am mildly ill due to the virus	13	2.4
I was/am moderately ill due to the virus	10	1.8
I was/am seriously ill due to the virus	3	0.6
I have sequelae as a result of the virus	10	1.8

Almost all participants (98.7%) reported at least one PTSD symptom, and the average IES-R total score was 30.77 (*SD* = 17.73). Avoidance symptoms presented the highest score (Table 2). Dividing the total scores by level, we observed that 37.4% showed normal, 18.6% mild, 6.3% moderate, and 37.8% severe affectations. The personal meaning average score was 107.79 (*SD* = 20.05). The area of meaning that presented the highest score was relationships, followed by achievement, and religion, the lowest.

**Table 2:** Means and standard deviations for IES-R and PMP-Brief

IES-R	*M*	*SD*	Min	Max
Intrusion	10.81	7.01	0	32
Avoidance	11.08	6.64	0	29
Hyperarousal	8.87	5.51	0	24
Total symptoms	30.78	17.73	0	82
PMP-Brief				
Achievement	16.23	3.62	4	21
Intimacy	15.85	5.37	3	21
Fair treatment	16.02	3.92	3	21
Relationship	16.69	3.80	3	21
Self-transcendence	16.19	3.79	3	21
Self-acceptance	15.74	3.67	3	21
Religion	11.03	6.74	3	21
Total meaning	107.79	20.05	42	147

### Pandemic Experience and PTSD Symptoms

Pearson correlations revealed that all PTSD symptoms were significantly associated with the number of life affectations (*r* = from 0.380 to 0.430, *p* < 0.001). Additionally, the independent samples *t*-test results showed many differences when comparing specifically on affected areas of life (Table 3). All symptoms were higher in individuals with academic, economic, couple, mental, and physical affectations. Persons who reported work affectations, had significantly more avoidance and hyperarousal symptoms. In addition, persons with social affectations only displayed higher intrusion and hyperarousal symptoms. Moreover, results showed many significant differences in the individuals’ personal experiences with the COVID-19 (Table 4). First, those who lost a close person due to the coronavirus and persons with suspected COVID-19, showed significantly higher PTSD symptoms. Second, persons who spent confinement alone reported significantly less intrusion, hyperarousal and total symptoms (Table 5). Lastly, Pearson’s correlation analyses revealed a significant positive association between home confinement duration and hyperarousal and reactivity symptoms (*p* = 0.005,* r* = 0.122) and negative associations between all symptoms and confinement assessment (*r* = from -0.445 to -0.342, *p* < 0.001).

**Table 3:** Comparison between IES-R symptoms on life affectations

Life affectations		IES-R symptoms					
		Intrusion, *M* (*SD*)	*t (sig)*	Avoidance, *M* (*SD*)	*t (sig)*	Hyperarousal, *M* (*SD*)	*t (sig)*
Work	Yes (n = 304)	11.32 (7.03)	1.888	11.66 (6.66)	2.311*	9.38 (5.55)	2.461*
	No (n = 239)	10.18 (6.95)		10.34 (6.54)		8.23 (5.41)	
Academic	Yes (n = 133)	12.51 (7.37)	3.228**	13.22 (6.67)	4.336**	10.25 (5.45)	3.330**
	No (n = 410)	10.27 (6.81)		10.39 (6.49)		8.43 (5.47)	
Economic	Yes (n = 274)	12.12 (6.92)	4.448**	12.16 (6.58)	3.879**	9.80 (5.51)	3.970**
	No (n = 269)	9.49 (6.87)		9.98 (6.52)		7.94 (5.36)	
Social	Yes (n = 353)	11.31 (6.90)	2.223*	11.45 (6.58)	1.748	9.29 (5.43)	2.401*
	No (n = 190)	9.91 (7.14)		10.41 (6.70)		8.11 (5.59)	
Couple	Yes (n = 149)	12.98 (7.58)	4.489**	12.86 (6.36)	3.882**	10.92 (5.51)	5.444**
	No (n = 394)	10.00 (6.61)		10.41 (6.62)		8.10 (5.32)	
Mental	Yes (n = 260)	14.23 (7.00)	12.264**	14.18 (6.18)	11.658**	11.93 (5.02)	14.553**
	No (n = 283)	7.69 (5.38)		8.23 (5.70)		6.07 (4.33)	
Physical	Yes (n = 196)	12.84 (7.00)	5.166**	12.87 (6.22)	4.815**	10.52 (5.35)	5.332**
	No (n = 347)	9.68 (6.77)		10.07 (6.66)		7.95 (5.39)	

^*^p < 0.05, **p < .001

**Table 4:** Pandemic features differences on PSTD symptoms

IES-R	Has lost a close person		*t*	*Sig*	*d*
	Yes, *n* = 97	No, *n* = 446			
	*M* (*SD*)	*M* (*SD*)			
Intrusion	13.53 (7.48)	10.23 (6.77)	-4.257	.000	6.908
Avoidance	12.52 (6.50)	10.77 (6.63)	-2.354	.019	6.614
Hyperarousal	11.16 (5.66)	8.38 (5.36)	-4.590	.000	5.418
Total	37.21 (18.45)	29.38 (17.28)	-3.992	.000	17.497
IES-R	Suspects having the virus		*t*	*Sig*	*d*
	Yes, *n* = 91	No, *n* = 452			
	*M* (*SD*)	*M* (*SD*)			
Intrusion	12.19 (6.31)	10.54 (7.12)	-2.043	.041	6.996
Avoidance	13.02 (6.57)	10.69 (6.59)	-3.077	.002	6.590
Hyperarousal	10.44 (5.17)	8.56 (5.53)	-2.983	.003	5.478
Total	35.65 (16.63)	29.80 (17.80)	-2.890	.004	17.617

**Table 5:** Differences on PTSD symptoms between alone and accompanied persons

IES-R	Spent confinement alone		*t*	*Sig*	*d*
	Yes, *n* = 43	No, *n* = 500			
	*M* (*SD*)	*M* (*SD*)			
Intrusion	8.42 (6.83)	11.03 (7.00)	2.348	.019	6.988
Avoidance	9.37 (6.50)	11.23 (6.63)	1.764	.078	6.628
Hyperarousal	6.95 (4.82)	9.04 (5.54)	2.392	.017	5.493
Total	24.74 (16.34)	31.30 (17.71)	2.335	.020	17.66

### Pandemic Experience and Meaning in Life

Moreover, the number of affectations and the confinement assessment positively correlated with all meaning in life dimensions (*r* = from -0.302 to -0.106, *p* < 0.01). Persons who reported economic affectations showed significantly lower fair treatment and self-acceptance (Table 6). Individuals with couple affectations had lower scores in fair treatment, self-transcendence and religion. All areas of meaning in life presented lower scores on individuals who reported mental affectations. Impaired physical health implied lower scores in all meaning dimensions except for achievement and intimacy. There were no differences in meaning in life between individuals with and without social impairments.

**Table 6:** Differences on meaning in life between individuals with and without life affectations

Life affectation		PMP-Brief													
		Achiev *M* (*SD*)	*t (sig)*	Intimacy *M* (*SD*)	*t (sig)*	FT *M* (*SD*)	*t (sig)*	RS *M* (*SD*)	*t (sig)*	ST *M* (*SD*)	*t (sig)*	SA *M* (*SD*)	*t (sig)*	Religion *M* (*SD*)	*t (sig)*
Work	Yes (N = 304)	16.17 (3.61)	-.519	15.55 (5.31)	-1.492	15.61 (3.94)	-2.812*	16.85 (3.58)	1.017	15.93 (3.91)	-1.872	15.55 (3.69)	-1.443	10.94 (6.57)	-.361
	No (n = 239)	16.33 (3.64)		16.24 (5.31)		16.55 (3.83)		16.51 (4.07)		16.54 (3.60)		16.00 (3.64)		11.15 (6.96)	
Academic	Yes (n = 133)	15.95 (3.98)	-1.042	14.14 (5.84)	-4.285**	14.92 (4.23)	-3.786**	15.87 (4.24)	-2.896**	15.82 (4.03)	-1.323	14.87 (3.93)	-3.188	9.36 (6.32)	-3.330**
	No (n = 410)	16.33 (3.50)		16.40 (5.10)		16.38 (3.74)		16.97 (3.62)		16.32 (3.70)		16.03 (3.52)		11.58 (6.78)	
Economic	Yes (n = 274)	16.00 (3.71)	-1.555	15.41 (5.50)	-1.956*	15.31 (3.91)	-4.317**	16.57 (3.81)	-.794	15.91 (4.01)	-1.770	15.23 (3.77)	-.3.344**	11.04 (6.71)	-.001
	No (n = 269)	16.48 (3.52)		16.30 (5.20)		16.74 (3.79)		16.83 (3.80)		16.49 (3.53)		16.28 (3.49)		11.04 (6.77)	
Social	Yes (n = 352)	16.10 (3.65)	-1.205	15.59 (5.49)	-1.514	16.09 (3.96)	.555	16.66 (3.71)	-.316	16.17 (3.74)	-.203	15.53 (3.66)	-1.863	10.73 (6.59)	-1.430
	No (n = 190)	16.49 (3.55)		16.33 (5.12)		15.89 (3.82)		16.77 (3.99)		16.24 (3.87)		16.15 (3.67)		11.60 (6.99)	
Couple	Yes (n = 149)	16.05 (3.71)	-.760	15.20 (5.12)	-1.735	15.15 (4.01)	-3.199**	16.20 (3.47)	-1.872	15.94 (3.73)	-.973	15.00 (3.71)	-2.936*	10.01 (6.48)	-2.197*
	No (n = 394)	16.31 (3.57)		16.10 (5.45)		16.35 (3.84)		16.89 (3.91)		16.29 (3.81)		16.03 (3.62)		11.43 (6.80)	
Mental	Yes (n = 260)	15.89 (3.66)	-2.146*	15.24 (5.39)	-2.558*	14.95 (4.09)	-6.300**	16.03 (3.93)	-3.964**	15.55 (4.08)	-3.838**	15.03 (3.80)	-4.431**	9.86 (6.45)	-3.959**
	No (n = 283)	16.56 (3.56)		16.41 (5.29)		17.00 (3.48)		17.31 (3.58)		16.79 (3.40)		16.41 (9.86)		12.12 (6.82)	
Physical	Yes (n = 196)	15.94 (3.93)	-1.429	15.35 (5.64)	-1.645	14.84 ( 4.39)	-5.433**	15.77 (4.19)	-4.358**	15.71 (4.15)	-2.239*	14.75 ( 3.77)	-4.852**	9.78 (6.30)	-3.307**
	No (n = 347)	16.41 (3.34)		16.14 (5.20)		16.69 (3.45)		17.22 (3.47)		16.47 (3.54)		16.31 (3.49)		11.75 (6.88)	

*p < .005, **p < .001. *FT *, fair treatment; *RS*, relationship; *ST*, self-trascendence; *SA*, self-acceptance

The independent samples *t*-test results indicated that those who lost a close person due to the coronavirus showed lower fair treatment scores and higher religion scores (Table 7). Furthermore, persons with suspected COVID-19 presented significantly lower fair treatment and religion. Lastly, persons who were severely ill (*M* = 16.21, *SD* = 3.61) in contrast to individuals that were not (*M* = 21, *SD* = 0.00), had significantly lower scores only in achievement (*r* = 2.290, *p* = 0.022). Regarding the confinement circumstances, individuals who were alone during the home lockdown had significantly lower scores in intimacy, religion, and total meaning (Table 8). All meaning in life dimensions showed significant and positive associations with the confinement assessment (*r* = from 0.136 to 0.352, *p* ≤ 0.001) and the assessment of company during confinement (*r* = from 0.145 to 0.404, *p* < 0.001).

**Table 7:** Pandemic features differences on personal meaning in life

PMP-Brief	Has lost a close person		*t*	*Sig*	*d*
	Yes, *n* = 97	No, *n* = 446			
	*M* (*SD*)	*M* (*SD*)			
Achievement	16.31 (3.92)	16.22 (3.56)	.209	.834	3.628
Intimacy	16.41 (5.09)	15.73 (5.43)	1.136	.257	5.373
Fair treatment	15.30 (3.84)	16.18 (3.92)	-2.010	.045	3.909
Relationship	16.39 (3.85)	16.76 (3.80)	-.873	.383	3.811
Self-transcendence	16.52 (3.96)	16.13 (3.75)	.913	.362	3.791
Self-acceptance	15.52 (4.09)	15.80 (3.58)	-.686	.493	3.677
Religion	12.68 (6.74)	10.68 (6.69)	2.664	.008	6.704
Total	109.12 (20.62)	107.50 (19.94)	.721	.471	20.068
PMP-brief	Suspects having the virus		*t*	*Sig*	*d*
	Yes, *n* = 91	No, *n* = 452			
Achievement	15.76 (3.33)	16.34 (3.66)	-1.389	.165	3.622
Intimacy	14.88 (5.58)	16.05 (5.31)	-1.895	.059	5.361
Fair treatment	14.89 (4.02)	16.25 (3.86)	-3.042	.002	3.891
Relationship	16.48 (4.00)	16.74 (3.77)	-.588	.557	3.812
Self-transcendence	15.67 (3.84)	16.30 (3.77)	-1.455	.146	3.786
Self-acceptance	15.13 (3.48)	15.87(3.70)	-1.755	.080	3.668
Religion	8.76 (6.54)	11.50 (6.69)	-3.572	.000	6.669
Total	101.57 (18.76)	109.04 (20.09)	-3.271	.001	19.882

**Table 8:** Independent samples t-test on PMP comparing between alone and accompanied persons

PMP-Brief	Spent confinement alone		*t*	*Sig*	*d*
	Yes, *n* = 43	No, *n* = 500			
Achievement	16.89 (3.59)	16.19 (3.62)	1.171	.242	3.623
Intimacy	11.70 (5.32)	16.21 (5.23)	-5.418	.000	5.239
Fair treatment	15.86 (3.98)	16.04 (3.91)	-.287	.778	3.924
Relationship	16.35 (4.54)	16.73 (3.74)	-.626	.532	3.812
Self-transcendence	16.26 (4.20)	16.19 (3.75)	.106	.916	3.794
Self-acceptance	15.81 (3.86)	15.74 (3.66)	.123	.902	3.678
Religion	8.21 (6.71)	1128 (6.71)	-2.886	.004	6.696
Total	101.05 (19.89)	108.37 (19.98)	-2.307	.021	19.980

### PTSD and Personal Meaning in Life

The results of Pearson's correlations showed that all areas of personal meaning in life (except for religion) were significantly and negatively correlated with IES-R symptoms (Table 9).

**Table 9:** Pearson correlations among PTSD symptoms and meaning dimensions

PMP-Brief	Intrusion		Avoidance		Hyperarousal		Total	
	*r*	*p*	*r*	*p*	*r*	*p*	*r*	*p*
Achievement	-.104	.016	-.128	.003	-.119	.006	-.126	.003
Intimacy	-.135	.002	-.152	.000	-.138	.001	-.153	.000
Fair treatment	-.298	.000	-.246	.000	-.297	.000	-.303	.000
Relationship	-.180	.000	-.189	.000	-.185	.000	-.199	.000
Self-transcendence	-.114	.008	-.139	.001	-.157	.000	-.146	.001
Self-acceptance	-.255	.000	-.175	.000	-.252	.000	-.245	.000
Religion	.001	.988	-.004	.923	-.026	.540	-.009	.825
Total	-.215	.000	-.208	.000	-.237	.000	-.236	.000

### Regression Analysis with Total PTSD Symptoms

Finally, a regression analysis using total IES-R score as dependent variable revealed that four aspects significantly explained higher PTSD symptoms: a greater number of affected areas of life, having lost a close person due to the coronavirus disease, and worse evaluations about the lockdown in general and the company during lockdown (Table 10).

**Table 10:** Multiple linear regression analysis with total PTSD symptoms as dependent variable

	Unstandardized coefficients		Standardized coefficients			95% Confidence interval for B	
	*B*	*SE*	Beta	*t*	*Sig*	Lower Bound	Upper Bound
Sex	1.583	1.606	.037	.986	.325	-1.572	4.738
Age	.058	.052	.041	1.123	.262	-.043	.160
Home lockdown duration	.014	.019	.027	.752	.453	-.023	.051
Alone/with others	2.782	2.511	.042	1.108	.269	-2.152	7.715
Confinement assessment	-4.143	.791	-.235	-5.240	.001	-5.695	-2.590
Company assesment	-2.303	.854	-.118	-2.695	.007	-3.981	-.624
Number of affected areas	2.731	.437	.260	6.256	.001	1.873	3.588
Know someone who died	4.948	1.706	.107	2.900	.004	1.596	8.301
Suspects having the virus	3.173	1.748	.067	1.815	.070	-.262	6.608
PMP achievement	-.115	.260	-.023	-.441	.659	-.626	.396
PMP fair treatment	-.267	.284	-.059	-.938	.348	-.826	.292
PMP relationship	-.188	.285	-.040	-.659	.510	-.748	.372
PMP self-trascendence	.440	.274	.094	1.605	.109	-.099	.979
PMP self-acceptance	-.373	.264	-.077	-1.412	.159	-.891	.146
PMP religion	.335	.175	.127	1.915	.056	-.009	.679
PMP total score	-.030	.141	-.034	-.213	.832	-.307	.247

## Discussion

The present findings confirm that traumatic symptomatology is a frequent consequence during the first months of the COVID-19 pandemic and its related affectations and confinement measures. In addition, our results cast a new light on how this experience and its psychological consequences are related to the personal meaning in life.

As expected, some specific characteristics of the personal pandemic experience were associated with higher PTSD symptoms. First, results showed that most of life affectations, and a higher total number of affectations, presented an association with PTSD symptoms. In addition, all PTSD symptoms were higher in individuals with diverse life affectations (academic, economic, couple, mental, and physical). This confirms the importance of the negative impact of affectations in functioning for the process of traumatic experiences (Rodríguez et al., 2012).

Second, the personal proximity to the virus. In the present study, those who lost a close person from COVID-19 showed more symptoms, confirming a severe problem related to the experience of grief and trauma (Eisma et al., 2019; Silove et al., 2017) and confirming that a close person’s unexpected death is associated with an increased incidence of following PTSD (Keyes et al., 2014). Besides, PTSD symptoms being higher in individuals who had the suspicion of having COVID-19 disease (but did not know for sure) corroborates significant psychological distress associated with ambiguous and uncertain situations a threat of infection (Taha et al., 2014). Third, concerning the lockdown features, the positive relationship between its duration and hyperarousal and reactivity symptoms (characterized by increased sensitivity and excessive reactions) suggests that the extended restriction caused individuals to constantly “feel on edge” and be more aware of threats. This entails alarming individual and collective psychosocial implications since hyperarousal may predict PTSD sequelae, future anxiety, and affective disorders, in addition to poorer quality of life (Blunt, 2016).

The company during lockdown was a crucial component of the experience. Despite the proof of loneliness’s adverse effects on physical and psychological health (Bzdok & Dunbar, 2020; Field et al., 2020; Walsh, 2020), our findings indicate disadvantageous circumstances (more severe PTSD symptoms and a worse subjective experience) for persons who were accompanied during confinement. There are two possible explanations for individuals reporting worse lockdown experiences and higher symptoms. First, the forced and unavoidable coexistence of family members or roommates, and the distress caused by a challenging situation, may have accentuated or increased previous conflicts. Moreover, an increment in domestic violence has been reported as a consequence of the forced coexistence due to confinement (Morgan and Boxall, 2020; Arenas-Arroyo et al., 2020). Second, the present results go along with the notion of adaptive loneliness, which happens from a positive transformation through embracing existential suffering and anxiety (Wong, 2020).

Furthermore, the pandemic’s personal experience was associated with some areas of personal meaning in life. Various areas of life affectations implied lower scores in meaning in life dimensions, especially those related to mental health. These findings support that the shattered assumptions of life, death, safety, and certainty would provide a need to restore order, meaning, and purpose (Janoff-Bulman, 1992) and that meaning in life is linked to psychological health and well-being (Brassai et al., 2011; Demirbas-Çelik, 2018; Li et al., 2019). In addition to perceiving less justice from life and society, people with economic affectations showed less self-acceptance. Negative external situations may influence own perceptions, in this case, especially about accepting personal limitations and the suffering itself. Regarding the proximity to the virus, persons who lost a close person showed had lower scores in fair treatment, meaning an increased perception of unfairness from society and life. Social restrictions might explain this since they impeded saying goodbye or carrying out meetings such as funerals, mourning, and mutual support (Imber-Black, 2020). Moreover, severely ill persons showed lower achievement scores, suggesting a significant affectation on the individual’s life priorities, putting aside the striving for and attaining significant life goals. Previous findings indicate that the positive experiences may cause beneficial psychological and stress-related outcomes in the context of the pandemic that individuals had during lockdown (Vázquez et al., 2021). The positive association of home confinement assessment with personal meaning of life indicates that people who had a better experience in confinement had a better chance of obtaining psychological benefits from the experience, such as reflecting on the meaning of life.

Furthermore, consistent with past research about the connection between meaning in life and better or positive posttraumatic outcomes (Weber et al., 2020), our results show it is inversely associated with PTSD symptomatology. This suggests that a meaningful life may prevent negative traumatic consequences and act as a tool for coping with difficult and unexpected experiences, since it plays a substantial role in reducing psychological suffering and allowing individuals to recover from trauma (Steger, 2012).

Among the variables that explained the symptoms of PTSD, some coincide with previous results, such as having a greater number of impaired areas of life (Evren et al., 2011) and experiencing the sudden and unexpected loss of a close person (Keyes et al., 2014). In addition, experiencing worse subjective lockdown circumstances (including the home lockdown in general and being alone or with someone else) also explained higher symptomatology, highlighting the need for psychosocial aids for persons affected by these specific features and the need to explore them further (Galea et al., 2020). Since meaning in life did not explain PTSD symptoms, we assume that when participants answered the survey, they were through an unknown and ongoing impact and focused their urges on a more fundamental coping (in this case, related to the physical illness, the lockdown, and current life affectations), which may have limited their time and energy to elaborate profound reflections on other concerns such as their meaning in life.

### Limitations

This study must be interpreted in light of the following limitations. First, participants were not selected via random sampling. Thus, we ignore the extent to which it represents the general population. Additionally, there was limited access to information. The internet-based data collection made the survey only accessible to people with an internet connection, filtering more marginalized or affected persons. For this reason, future research should include diverse populations and socioeconomic conditions. Secondly, due to time concerns, this is an exploratory and preliminary study, and its cross-sectional design impedes us from determining causes and effects. Moreover, further research should re-evaluate meaning in life in conjunction with the concept of pandemic fatigue, given that it has been more than a year since confinement and thus a more extended period of change and reflection.

## Conclusion

The COVID-19 pandemic, the experience of confinement measures, and the related multiple crises are substantial psychological stressors that will continue striking human beings for an indefinite time. In particular, persons with academic, economic, couple, mental, and physical affectations due to the pandemic, individuals who have lost someone to the virus, persons diagnosed with the virus, and those with long and negative lockdown experiences may show more severe symptoms and distress. These findings have important implications that public health services may consider for courses of action to address and monitor psychosocial needs and provide mental health support for affected individuals, particularly those with severe hyperarousal and reactivity symptoms. Moreover, educational and health services need to address the different aspects involved in meaning in life, such as developing training and intervention programs that include specific objectives such as working on: (a) interpersonal relationships, trust, reciprocity, and cooperation, (b) short and long term life goals and identifying the phases for their achievement, (c) personal limitations, frustration, and suffering, (d) life transcendence and its implications in the common well-being. Future studies need to be done on both home conflicts during the lockdown and alternative positive outcomes of the pandemic experience.

## Data Availability

Authors can confirm that all relevant data are included in the article.
